# Functionalization of Magnetic Nanoparticles by Folate as Potential MRI Contrast Agent for Breast Cancer Diagnostics

**DOI:** 10.3390/molecules25184053

**Published:** 2020-09-04

**Authors:** Hamid Heydari Sheikh Hossein, Iraj Jabbari, Atefeh Zarepour, Ali Zarrabi, Milad Ashrafizadeh, Afrooz Taherian, Pooyan Makvandi

**Affiliations:** 1 Department of Biotechnology, Faculty of Biological Sciences and Technology, University of Isfahan, Isfahan 81746-73441, Iran; Hamidheydari93@gmail.com (H.H.S.H.); atefeh.zarepour@gmail.com (A.Z.); 2 Faculty of Physics, University of Isfahan, Isfahan 81746-73441, Iran; i_jabbari@yahoo.com (I.J.); Afrooztaherian@gmail.com (A.T.); 3 Sabanci University Nanotechnology Research and Application Center (SUNUM), Tuzla, 34956 Istanbul, Turkey; 4 Department of Basic Sciences, Faculty of Veterinary Medicine, University of Tabriz, Tabriz 51666-16471, Iran; dvm.milad73@yahoo.com; 5 Istituto Italiano di Tecnologia, Centre for Micro-BioRobotics, viale Rinaldo Piaggio 34, 56025 Pontedera, Pisa, Italy; 6 Department of Medical Nanotechnology, Faculty of Advanced, Technologies in Medicine, Iran University of Medical Sciences, Tehran 14496-14535, Iran

**Keywords:** contrast agents, MRI, polyglycerol, silica nanoparticles, SPION

## Abstract

In recent years, the intrinsic magnetic properties of magnetic nanoparticles (MNPs) have made them one of the most promising candidates for magnetic resonance imaging (MRI). This study aims to evaluate the effect of different coating agents (with and without targeting agents) on the magnetic property of MNPs. In detail, iron oxide nanoparticles (IONPs) were prepared by the polyol method. The nanoparticles were then divided into two groups, one of which was coated with silica (SiO_2_) and hyperbranched polyglycerol (HPG) (SPION@SiO_2_@HPG); the other was covered by HPG alone (SPION@HPG). In the following section, folic acid (FA), as a targeting agent, was attached on the surface of nanoparticles. Physicochemical properties of nanostructures were characterized using Fourier transform infrared spectroscopy (FT-IR), transmission electron microscopy (TEM), and a vibrating sample magnetometer (VSM). TEM results showed that SPION@HPG was monodispersed with the average size of about 20 nm, while SPION@SiO_2_@HPG had a size of about 25 nm. Moreover, HPG coated nanoparticles had much lower magnetic saturation than the silica coated ones. The MR signal intensity of the nanostructures showed a relation between increasing the nanoparticle concentrations inside the MCF-7 cells and decreasing the signal related to the *T_2_* relaxation time. The comparison of coating showed that SPION@SiO_2_@HPG (with/without a targeting agent) had significantly higher *r*_2_ value in comparison to Fe_3_O_4_@HPG. Based on the results of this study, the Fe_3_O_4_@SiO_2_@HPG-FA nanoparticles have shown the best magnetic properties, and can be considered promising contrast agents for magnetic resonance imaging applications.

## 1. Introduction

Because of their unique physicochemical properties, nanoparticles (NP) have broad applications in a variety of biomedical researches, like implants [1], drug delivery systems [2], tissue engineering [3], and infection therapy [4]. One of the significant applications of nanomaterials in medicine is the timely detection and treatment of cancer [5]. Different nanoprobes such as quantum dots have been developed for the early diagnosis of breast cancer, and this requires understanding the distribution and removal of nanoparticles at the breast tumor site [6]. One of the potential applications of NP is in diagnostic medicine, particularly for magnetic resonance imaging (MRI).

Briefly, MRI is a noninvasive imaging modality that, by exposing the hydrogen protons to an external magnetic field (B_0_), leads to aligning some parts of protons towards B_0_ direction and then rotation around it. Then, radiofrequency (RF) waves apply in the equal frequency with the precession of the protons, resulting in the RF pulse energy adding to the protons. Subsequently, when the RF pulse is turned off, protons flip back to the original state and generate a radiofrequency signal in a process called “relaxation” [7,8,9].

Two kinds of relaxation processes exist; namely, longitudinal or *T*_1_ relaxation, also called spin-lattice relaxation, and transverse or *T*_2_ relaxation, referred to as spin-spin relaxation. The former refers to the process through which the net longitudinal magnetization goes back to its earliest maximum value parallel to the magnetic field, as described in the following equation [8]:

(1) Mz=Mz,0(1−e−tT1)

where *M_z_*_,0_ refers to the magnetization in an equilibrium state, and *T*_1_ stands for the time for *M_z_* to reach 63% of the original magnetization after the application of the RF pulse.

The latter is the process referring to the transverse components of magnetization decay or dephases, as described in the following equation [8]:

(2) Mxy=Mxy,0e−tT2

in which *M_xy_* illustrates the decay of magnetization, *M_xy_*_,0_ depicts the initial transverse magnetization, and *T*_2_ shows the time for transversal magnetization dropping to 37%.

Although MRI has gained much publicity in medicine diagnostics, there are some drawbacks associated with it. One of the most important ones is low sensitivity that reduces its potential in molecular level detection [10]. To overcome this restriction, increasing contrast between healthy and diseased tissues is of great importance.

Typically, elements like gadolinium, iron, and manganese that possess unpaired electron spins are referred to as *T*_1_ contrast agents. They could increase the *T*_1_ relaxation time via increasing the signal intensity, thus causing a positive contrast in images. On the other hand, superparamagnetic iron oxide nanoparticles (SPIONs) decrease the signal intensity via the reduction of *T*_2_ relaxation time, resulting in images with negative contrast [11,12,13,14]. Gadolinium is the most widely used contrast agent in MRI, which has many limitations, the most important of which are its cytotoxicity and non-biodegradability. Recently, SPIONs have attracted more attention due to their effectiveness at low concentration, biocompatibility, and easy functionalization that make them suitable alternatives for gadolinium in MRI [15,16].

Apart from their use as MRI contrast agents, iron oxide nanoparticles have several other potential applications, like drug delivery vehicle, cell tracking, tissue engineering, separation of biomolecules, and immobilization of enzymes or proteins in biomedical engineering [17]. However, there are obstacles and challenges for using SPIONs, including their tendency to aggregation and the protein corona effect [10,11,12,14]. One of the most prevalent approaches used to meet these obstacles is coating the surface of nanoparticles with polymers [18,19].

Hyperbranched polyglycerol (HPG) is an amphiphilic biocompatible polyether polyol with a high degree of branching, a dense structure, and a large number of terminal hydroxyl groups which can provide a broad area for encapsulation of different types of drugs and attachment of targeting molecules. Coating the nanoparticles with HPG provides them with suitable properties, such as increasing their water solubility, biocompatibility, and protein resistance ability, which are comparable to polyethylene glycol (PEG) [20,21]. Moreover, HPG polymers possess high thermal and oxidative stability [22,23].

One of the drawbacks of nanocarriers is their cytotoxicity against normal cells. This problem can be overcome using biocompatible polymers [24,25]. Furthermore, coating nanoparticles can delay their clearing from the body, and improve stability [26]. Silica is extensively used for coating nanoparticles to, in one hand, prevent their aggregation, and on the other hand, support against oxidation. It is held that silica can promote the biocompatibility of nanoparticles. The interesting point is that silica is has a high affinity for binding into surface of nanoparticles [27]. The recently published articles have also confirmed the aforementioned statements, and benefits of using silica for coating nanoparticles [28].

In this research, the impact of different coatings on the $r_{2}$ relaxivity of MR imaging of iron oxide nanoparticles was investigated. For this purpose, iron oxide magnetic nanoparticles were synthesized using the polyol method. Next, for a comparison of coating effects, synthesized nanoparticles were coated with HPG and SiO_2_-HPG. Then, folic acid as targeting ligand was conjugated in a covalent bond to the terminal hydroxyl groups of coating nanoparticles. Different types of physicochemical analyses (like Fourier transform infrared spectroscopy (FT-IR), transmission electron microscopy (TEM), and vibrating sample magnetometer (VSM)) were used for the characterization of the nanoparticles. Moreover, the cytotoxicity of nanoparticles was determined using MTT colorimetric assay. Finally, the differences between the magnetic properties of the coated nanoparticles (with/without targeting agent) were investigated via MRI analysis.

## 2. Results

### 2.1. Synthesis and Characterization of Nanoparticles

**Figure 1:**
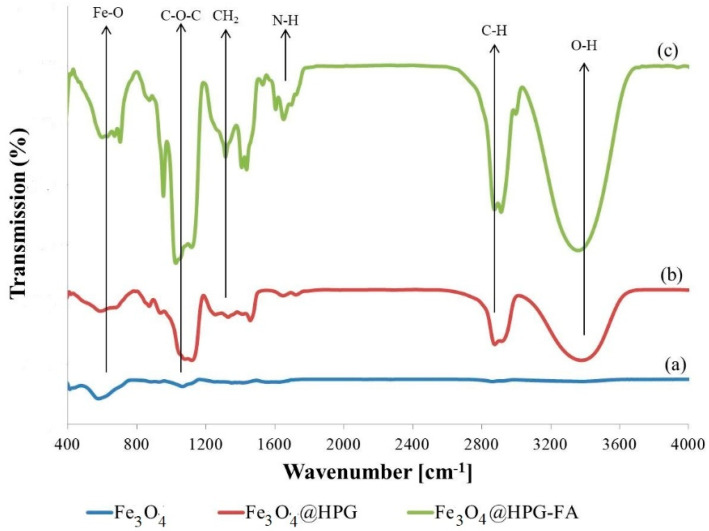
FT-IR spectroscopy of (**a**) Fe_3_O_4_ (**b**) hyperbranched polyglycerol (HPG) grafted Fe_3_O_4_ and (**c**) Fe_3_O_4_@HPG-FA.

**Figure 2:**
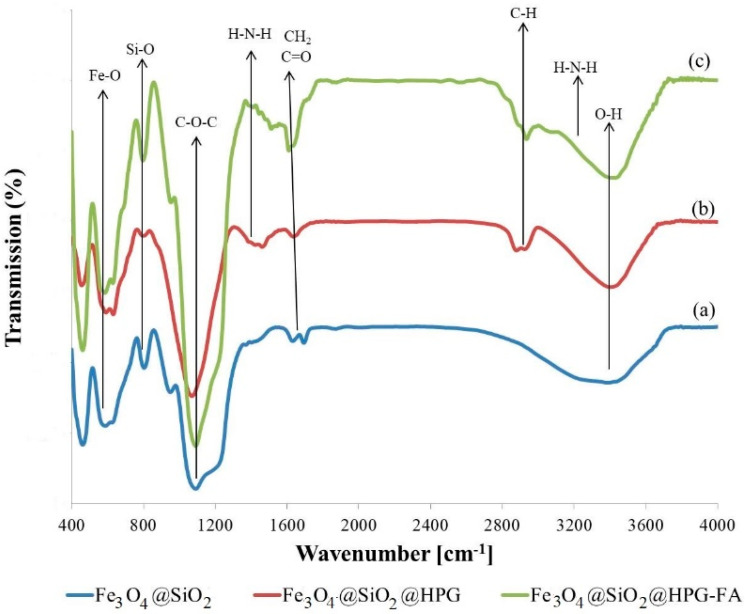
FT-IR spectroscopy of (**a**) Fe_3_O_4_@SiO_2_, (**b**) Fe_3_O_4_@SiO_2_@HPG, and (**c**) Fe_3_O_4_@SiO_2_@HPG-FA.

Fe_3_O_4_ nanoparticles were synthesized with success by using the polyol method, and then they were coated with SiO_2_ and HPG. The changes that occurred in the chemical structure of the nanoparticles were characterized by FT-IR spectroscopy (Figure 1 and Figure 2). Based on the result in Figure 1, the absorption peak at about 585 cm^−1^ belongs to the stretching vibration of Fe-O bonds in Fe_3_O_4_ nanoparticles, which could confirm the presence of Fe as the essential element in the nanoparticles. The characteristic band at around 1115–1050 cm^−1^ is associated with the C-O-C stretching, which could also reveal the conjugation of TREGs as an organic component on the particles surface (Figure 1a) [29,30,31].

Figure 1b illustrates the FTIR spectrum of nanoparticles coated by hyperbranched polyglycerol. The broad band at 3400 cm^−1^ is associated with the stretching vibration of the O-H bond, and the split peaks between 2800–2900 cm^−1^ belong to the C-H stretching (symmetric and asymmetric) of the polyglycerol. Moreover, the bands at around 1300–1500 cm^−1^ are associated with the bending vibration of the CH_2_ Groups. These results approve the polymerization of monomers on the surface of nanoparticles [16,21,30,32].

Figure 1c shows the FTIR result of Fe_3_O_4_ @HPG-FA nanoparticles. The intense peaks at 1400 and 1650 cm^−1^ correspond to the p-amino benzoic acid moieties and aromatic ring stretch of FA pteridine ring. Furthermore, significant broadening peaks at 1620 cm^−1^ and 1650 cm^−1^ represent the N-H groups of FA components, confirming the emergence of targeted SPION@HPG with FA [33,34].

In Figure 2a, the band at around 590 cm^−1^ is attributed to the vibration of the Fe-O bond, which shows the presence of Fe_3_O_4_ components. In addition, the bonds at about 1089 cm^−1^ and 806 cm^−1^ are associated with the Si-O vibration, revealing the presence of the SiO_2_ shell. As depicted in Figure 2b, the absorbance peaks at around 3435 cm^−1^ and 1080 cm^−1^ confirm the presence of HPG on the surface of nanoparticles , and they are attributed to the OH bond and C-O-C ether stretch bond, respectively [20,35].

The FTIR spectra of the FA grafted Fe_3_O_4_@SiO_2_@HPG nanoparticles illustrate peaks at 1610 and 1410 cm^−1^, which correspond to the aromatic ring stretch of pteridine ring and p-amino benzoic acid moieties of FA (Figure 2c). A significant broadening peak at 1610 cm^−1^ also corresponds to the ester linkage that is formed between folic acid and the hydroxyl groups. Moreover, the strong band at around 2912 cm^−1^ corresponds to the CH_2_ stretching vibrations [34,36,37].

**Figure 3:**
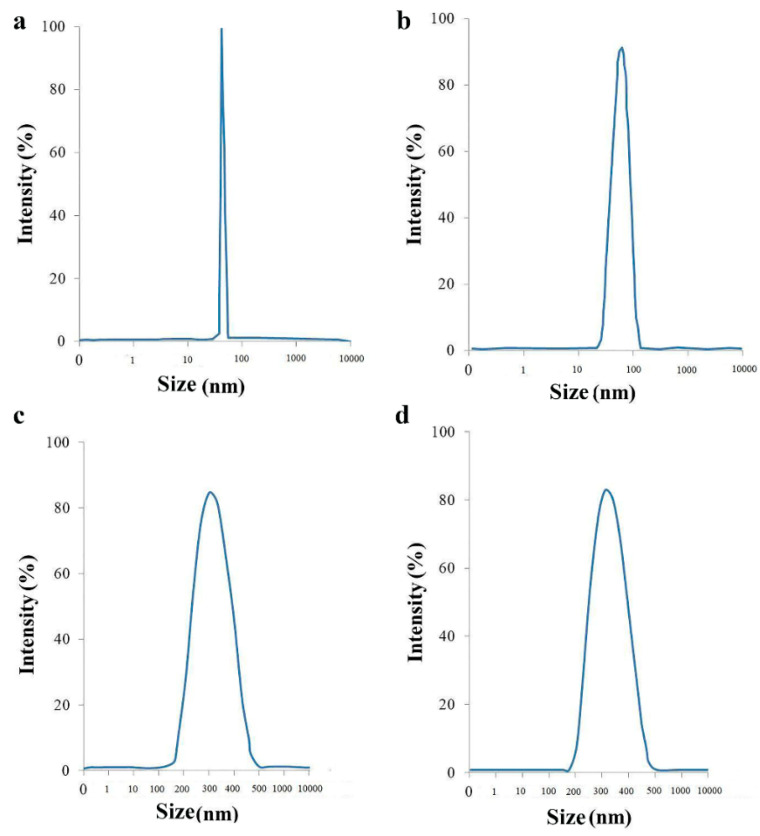
Size distribution analysis of (**a**) Fe_3_O_4_, (**b**) Fe_3_O_4_@HPG, (**c**) Fe_3_O_4_@SiO_2_, and (**d**) Fe_3_O_4_@SiO_2_@HPG.

Figure 3 shows the results of DLS analysis of synthesized and functionalized iron oxide nanoparticles after each step of surface modification. It can be seen that the synthesized Fe_3_O_4_ nanoparticles have a narrow size distribution with a mean of ~55 nm, whereas after each step of functionalization, the size increased due to the presence of new layers. In agreement with the literature, the size of the modified iron oxide is increased due to the fact that coating bring new layers to the surface of magnetic nanoparticles; accordingly, the size is enhanced [38,39].

**Figure 4:**
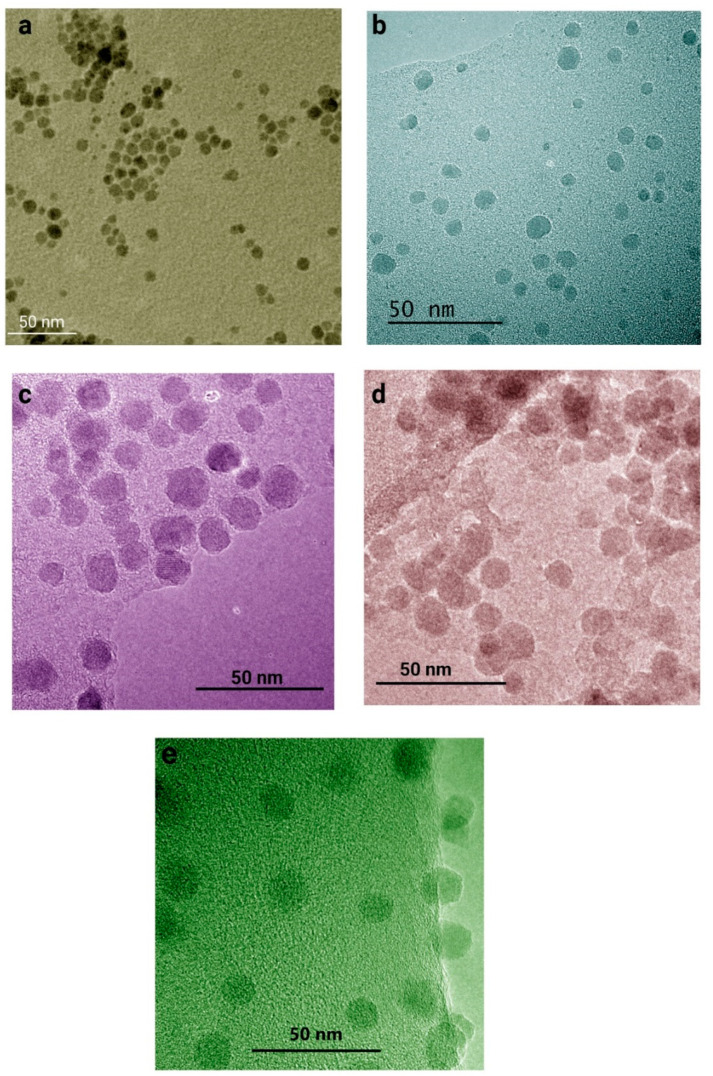
UHR-TEM analysis of (**a**) Fe_3_O_4_, (**b**) Fe_3_O_4_@HPG, (**c**) Fe_3_O_4_@SiO_2_, (**d**) Fe_3_O_4_@SiO_2_@HPG, and (**e**) Fe_3_O_4_@SiO_2_@HPG-FA.

The size and core-shell structure of nanoparticles were characterized by ultra-high-resolution transmission electron microscopy. For this test, Fe_3_O_4_, Fe_3_O_4_@HPG, Fe_3_O_4_@SiO_2_, Fe_3_O_4_@SiO_2_@HPG, and Fe_3_O_4_@SiO_2_@HPG-FA were characterized by TEM, as the results show in (Figure 4). In agreement with other literature studies [40,41], there is a difference between DLS and TEM sizes measurements which are normally attributed to the fundamental difference between intensity and number-weighted particle size distributions, and the differences between the dry and hydrodynamic radius of particles.

As is evident from TEM images, Fe_3_O_4_ nanoparticles (Figure 4a) have a spherical shape with a particle size of about 10 nm. Since the HPG cannot be observed in the TEM images, the size of HPG-grafted Fe_3_O_4_ nanoparticles is the same as that of bare nanoparticles. However, it could be seen that HPG coating positively affects the distribution of nanoparticles (Figure 4b). The Fe_3_O_4_@SiO_2_ nanoparticles show a distinct core-shell structure, and the size that is deduced from the TEM image (Figure 4c) is around 20 nm, which is smaller than Fe_3_O_4_@SiO_2_@HPG, with a size about 30 nm. From TEM results, we can reach the conclusion that the HPG coating conceals one core of Fe_3_O_4_, while several Fe_3_O_4_ cores are covered by silica coating, which leads to the formation of semi-composite structures.

It has been reported that polyglycerol can prevent the aggregation and enhance the stability of iron oxide nanoparticles, since these coatings have high hydrophilicity. Such stability (e.g., stabilizing the nanoparticles for about 2 months) has been shown in previous literature [38,39].

### 2.2. MTT Assay

**Figure 5:**
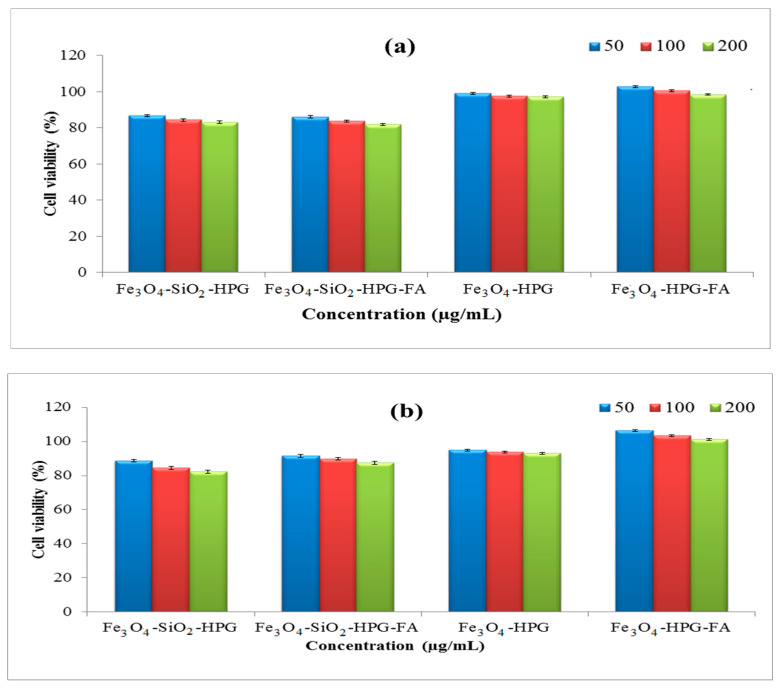
Cytotoxicity evaluation of nanoparticles after (**a**) 24 h and (**b**) 48 h on the MCF-7 cell line.

One of the most common tests used for the assessment of the impact of foreign materials on the viability of cells is MTT assay, which is based on the enzymatic reduction of MTT salt to the purple formazan brought about by the mitochondria of viable cells [33,42]. In this research, the impact of cytotoxicity of various concentrations of nanoparticles on the viability of the MCF-7 cell line was evaluated by MTT assay for two days, and the results of this test are shown in Figure 5. As is clear in the figures, both of the polymeric coated nanoparticles (with or without folic acid) have no significant effect on the viability of the cells. Moreover, the viability of cells exposed by the targeted samples was a little higher than the non-targeted ones. These results confirm the cytocompatibility of this carrier, which is a critical factor for utilizing the nanocarriers in vivo. Based on the literature, this feature is due to the presence of polyglycerol, which could provide high biocompatibility for nano drug delivery systems [43,44].

### 2.3. Value Stream Mapping (VSM)

**Figure 6:**
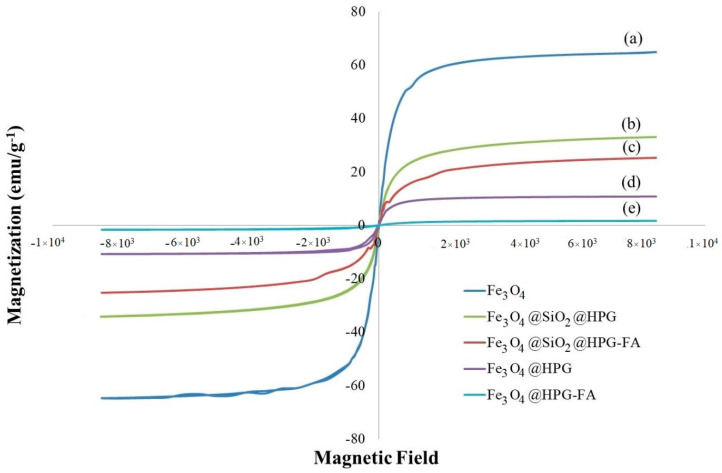
Magnetic behavior of (**a**) Fe_3_O_4_ nanoparticles (**b**) Fe_3_O_4_@SiO_2_@HPG (**c**) Fe_3_O_4_@SiO_2_@HPG-FA (**d**) Fe_3_O_4_@HPG (**e**) Fe_3_O_4_@HPG-FA.

The magnetic properties of Fe_3_O_4_, Fe_3_O_4_@HPG, Fe_3_O_4_@HPG-FA, Fe_3_O_4_@SiO_2_@HPG, and Fe_3_O_4_@SiO_2_@HPG-FA were measured at room temperature by employing VSM (Figure 6). As can be seen, all magnetization curves are s-shaped over the applied magnetic field, confirming that they all have superparamagnetic behavior. The saturation magnetization (M_s_) of Fe_3_O_4_ nanoparticles was decreased from 64.84 to 33.86, 25.80, 10.77, and 1.66 emu/g^−1^, when SiO_2_@HPG, SiO_2_@HPG-FA, HPG, and HPG-FA were grafted to them [16,45]. These results reveal that HPG coating could decrease M_s_ of nanoparticles more than SiO_2_@HPG coating, which may be due to its longer polymerization time leading to the production of a much thick layer on the surface of magnetic nanoparticles. By combing the results of VSM with TEM results, it could be said that Fe_3_O_4_@HPG nanoparticles contain one core of magnetic nanoparticle covered by a layer of branched polyglycerol. In contrast, Fe_3_O_4_@SiO_2_@HPG nanoparticles are composed of a composite of some magnetic nanoparticles dispersed in the SiO_2_ matrix and then covered by HPG. This event also could affect the magnetization property of the nanoparticles, leading to a reduction in the amount of magnetic saturation. M_s_ of about 7–22 emu/g^−1^ is usually adapted for biomedical and bioengineering applications; therefore, the Fe_3_O_4_@SiO_2_@HPG nanoparticles with higher M_s_ are acceptable for bio applications.

### 2.4. MR Imaging of Cells after They Are Incubated by Nanoparticles

**Figure 7:**
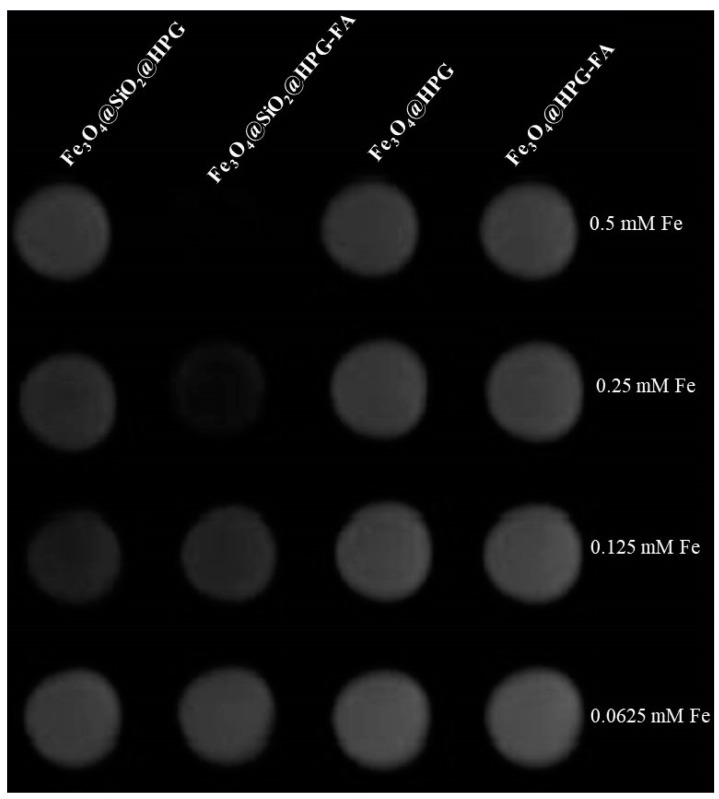
*T*_2_-weighted image of MCF-7 cells after they are treated with different concentrations Fe of each contrast agent.

The MRI was employed in order to evaluate the impact of coating on magnetic behavior of the nanoparticles used as contrast agent. For this test, Fe_3_O_4_@SiO_2_@HPG and Fe_3_O_4_@HPG with and without FA were used, and the results are described in Figure 7. According to this Figure, the MR signal intensity of the nanoparticles had a relationship with different concentrations of samples, so that by increasing the concentrations of nanoparticles inside the MCF-7 cells, the MR signal that is associated with the *T*_2_ relaxation time is significantly decreased, leading to a negative contrast in *T*_2_-weighted images.

**Figure 8:**
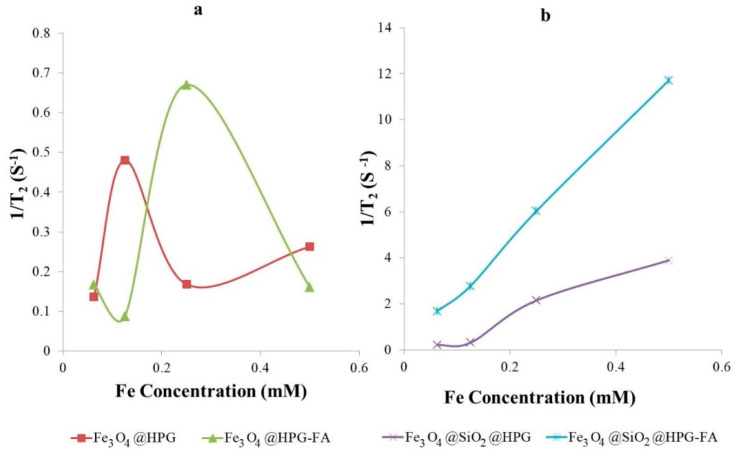
1/*T*_2_ values as a function of concentration of (**a**) Fe_3_O_4_@HPG and Fe_3_O_4_@HPG-FA; (**b**) Fe_3_O_4_@SiO_2_@HPG and Fe_3_O_4_@SiO_2_@HPG-FA.

The results revealed that due to their low magnetic saturation, HPG coated nanoparticles have low amounts of 1/*T*_2_ (0.5 and 0.7 for nanoparticles without and with FA, respectively), and are not suitable for MRI contrast agents (Figure 8a); while, by changing the coating of nanoparticles, the amounts of 1/*T*_2_ also changed, so that it increased from 4 S^−1^ for Fe_3_O_4_@SiO_2_@HPG to 12 S^−1^ for Fe_3_O_4_@SiO_2_@HPG-FA (Figure 8b). Generally, *T_2_* measurement of the samples indicated that the relaxation times (1/*T*_2_) increased much faster for nanoparticles targeted with folic acid than for those not targeted.

The results of $r_{2}$ of different nanoparticles are listed in Table 1. Based on the results in Table 1, SiO_2_@HPG coated nanoparticles have higher $r_{2}$ in contrast to HPG one. Moreover, targeted samples have significantly higher $r_{2}$ values in comparison to non-targeted ones due to the presence of high amounts of folate receptors on the surface of the MCF-7 cell line. When FA conjugated to Fe_3_O_4_@SiO_2_@HPG, the $r_{2}$ value was increased from 8.87 to 23.25 mM^−1^S^−1^ that confirmed the targeting role of FA. The results reveal a preferential uptake of Fe_3_O_4_@SiO_2_@HPG-FA nanoparticles by the cells overexpressing the folate receptor [46,47]. Furthermore, Fe_3_O_4_@SiO_2_@HPG-FA targeted nanoparticles have $r_{2}$ 23.25 mM^−1^S^−1^, which is higher than Fe_3_O_4_@HPG-FA with $r_{2}$ 0.131 mM^−1^S^−1^. This is related to the higher M_s_ of these nanoparticles in comparison to the Fe_3_O_4_@HPG-FA, and is associated with the high amounts of HPG coating on the Fe_3_O_4_@HPG surface, resulting in the reduction in the magnetic intensity of nanoparticles.

## 3. Discussion

Early detection is a critical factor for successful cancer therapy. In this regard, there are several methods for the early detection of cancer, among the most important of which is MRI, a highly sensitive method with low side effects. Since the possible side effects of the high magnetic field in MRI are still unknown, the clinical application of MRI with magnetic field less than 7T is FDA approved. Therefore, it is necessary to use a contrast agent, which could increase the sensitivity of MRI detection [10]. The unique properties of magnetic nanoparticles such as excellent biocompatibility and high magnetic behavior introduce them as an appropriate candidate for biomedical applications [48]. Consequently, the design and fabrication of biocompatible water-soluble iron oxide nanoparticles to be used as contrast agents have attracted a lot of attention, and significant attempts have been made in this field [49,50,51]. This study mainly aimed to compare the impact of two different coatings on the magnetic property of iron oxide nanoparticles using an MRI contrast agent.

Iron oxide magnetic nanoparticles were synthesized by using the polyol method. Then, the nanoparticles were coated with SiO_2_ and HPG, and folic acid as a targeting ligand was conjugated to the terminal hydroxyl groups of coating nanoparticles in a covalent manner. The findings of this study showed that coating Fe_3_O_4_ nanoparticles with SiO_2_ and HPG did not affect the viability of the MCF-7 cell line; while targeted nanoparticles, especially for the Fe_3_O_4_@HPG@FA, showed a partial cytotoxicity effect on the cells at high concentrations. This result has also been observed by other researchers [52]. The magnetic saturation was found at 33.86, 25.80, 10.77, and 1.66 emu/g^−1^ when HPG@SiO_2_, HPG@SiO_2_@HPG-FA, HPG, and HGP-FA were grafted to Fe_3_O_4_ nanoparticles, which were less than that of pure nanoparticles, with M_s_ of about 64.84 emu/g^−1^. These differences suggest that HPG coating could decrease M_s_ of nanoparticles more than SiO_2_@HPG. In addition, the reduction of nanoparticles’ magnetization due to the surface modifications has been confirmed by several studies [53,54].

Coating nanoparticles by HPG and SiO_2_@HPG produced an $r_{2}\;$value of 0.029 and 8.87 mM^−1^S^−1^, respectively. These results demonstrated that coating nanoparticles with HPG significantly decreased the magnetic intensity of nanoparticles, due to the high polymerization and fewer number of Fe_3_O_4_ in the core in comparison to the silica coating nanoparticles. These results indicate that changing the surface coatings of nanoparticles could influence the transversal relaxivities ($r_{2}$) [38]. That the relaxivity is a vital parameter for the estimation of the efficiency of a *T_2_* contrast agent is quite well known. Hajesmaeelzadeh et al. reported that, by increasing the thickness of coating on iron oxide nanoparticles, there was an increase in their relaxivity. Moreover, they showed that with an increase in the nanoparticles size, the amount of $r_{2}$ relaxivity decreased. This result confirms that the type and amount of the coating affect the $r_{2}$. values relaxivities [55]. According to Bitar et al., biocompatible SiO_2_ coating of nanoparticles makes them more dispersible in bio-media and easy to be functionalized using biomolecules for biomedicine research, too [56].

**Figure 9:**
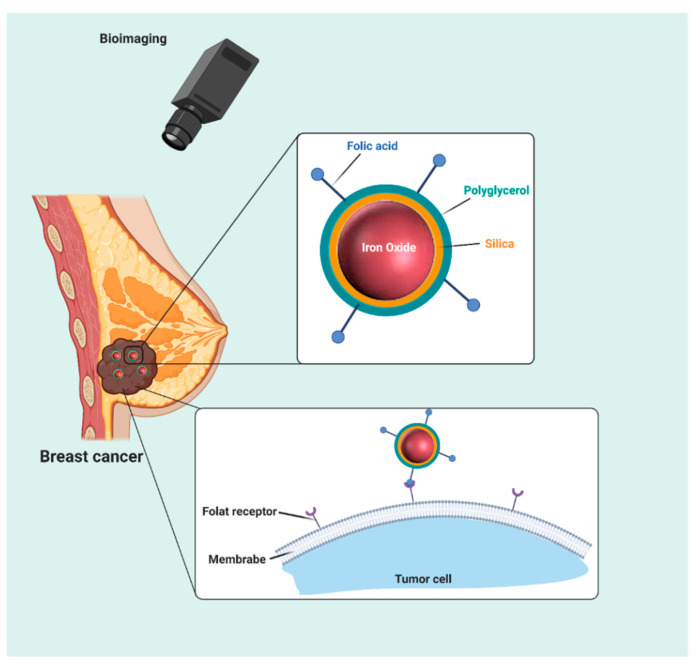
Schematic representation of using nanoparticles for selective targeting of breast cancer cells.

Furthermore, targeting the nanoparticles has an essential function in improving the particles selectivity against cancer cells. In addition, it could increase the accumulation of contrast agents needed for imaging in the targeted site [57]. The findings of this research reveal that the functionalization of HPG with FA leads to the attachment of nanoparticles to the folate receptors, which are expressed on the surface of MCF-7 cells that are later taken into the cell via receptor-mediated endocytosis (Figure 9). Nanoparticles lacking FA enter the MCF-7 cells using the non-specific penetration process. As a consequence, folic acid targeted nanoparticles have substantial $r_{2}$ relaxivities in comparison to the non-targeted one, just as what was reported in other studies [58,59]. As was predicted, Fe_3_O_4_@SiO_2_@HPG-FA nanoparticles, due to their higher magnetic intensity, have better $r_{2}$ relaxivities (23.25 mM^−1^S^−1^) in contrast to the Fe_3_O_4_@HPG targeted nanoparticles. Gholibegloo et al. reported that folic acid decorated magnetic nanoparticles have an $r_{2}$ value of about 54 mM^−1^S^−1^ [60]. However, they used cyclodextrin for the surface modification of nanoparticles as well as curcumin drug accompany with their MRI analysis, which could affect the MRI signal intensity. Moreover, when folic acid moieties were conjugated to the SiO_2_@HPG and HPG, the hydrophilicity of these nanoparticles also increased, leading to the elevation of their circulation time. Since particles with an $r_{2}$ value of more than 20 mM^−1^S^−1^ are well suited for the MRI contrast agent, Fe_3_O_4_@SiO_2_@HPG-FA with an $r_{2}$ value above 23 mM^−1^S^−1^, compared to non-targeting nanoparticles, possess more potential to be employed as contrast agents in MRI analysis.

## 4. Materials and Methods

### 4.1. Materials

Iron (III) acetylacetonate (Fe(acac)_3_), ethyl acetate, dicyclohexylcarbodiimide (DCC), and dimethyl aminopyridine (DMAP) were bought from Merck Co. (Munchen, Germany). Triethylene glycol (TREG) was obtained from Novachem (Calgary, Canada). Glycidol, dimethyl sulfoxide (DMSO), tetraethyl orthosilicate (TEOS), (3-Aminopropyl) triethoxysilane (APTES) and folic acid were bought from Sigma Aldrich Co. (St. Louis, MO, USA). For cell culture, the MCF7 cell line was obtained from Pasteur Institute (Tehran, Iran). Dulbecco’s modified Eagle’s medium (DMEM), phosphate buffer saline (PBS), and penamiicillin-streptomycin were purchased from BioIdia (Tehran, Iran), and fetal bovine serum (FBS) was obtained from Gibco Co. (Grand Island, NY, USA). 3-(4,5-dimethylthiazol-2)-2,5-diphenyltetrazolium bromide (MTT) was bought from Sigma Co. (Steinheim, Germany).

### 4.2. Fabrication of Fe_3_O_4_ Nanoparticles

Fe_3_O_4_ nanoparticles were prepared using the polyol method based on previous reports [61]. In summary, 0.053 g of Fe(acac)_3_ and 30 mL TREG were mixed using magnetic stirring. Then, the solution was slowly heated to 300 °C under N_2_ atmosphere, and it was kept at the reflux temperature for about 60 min. After cooling it to room temperature, 20 mL ethyl acetate was added to it while mixing the solution with a shaker. Then, nanoparticles were precipitated through a neodymium magnet and were washed with ethyl acetate three times, and the final precipitate was dried with a freeze-dryer (Vaco 5-ZIRBUS Tech, Harz, Germany) [62].

### 4.3. Synthesis of Polyglycerol Grafted Fe_3_O_4_

The anionic ring-opening polymerization of glycidol was carried out in accordance with the literature [33,62,63]. In brief, 1 mL of glycidol monomers was added to 30 mg Fe_3_O_4_ nanoparticles, and the mixture was sonicated at 25 °C for one h before being heated at 140 °C under N_2_ atmosphere for about 20 h. Then, it was cooled to room temperature, the blackish gel was poured into 10 mL DI water, and it was sonicated for 30 min. After dialysis, and the solution against DI-water (dialysis membrane 12 kDa) for 24 h, the nanoparticles were dried by freeze-dryer.

### 4.4. Fabrication of Fe_3_O_4_-Silica Core-Shell Nanoparticles

For other types of nanoparticles, Fe_3_O_4_ nanoparticles were coated by silica at first, and the silica-coated nanoparticles were functionalized by HPG. Briefly, the as-prepared Fe_3_O_4_ nanoparticles suspension was diluted with 60 mL of absolute ethanol with the help of sonication for 15 min, followed by the addition of 30 mL DI water and 25 mL NH_4_OH 28%. Then, 2 mL tetraethyl orthosilicate (TEOS) was added, and the mixture was handled by sonication at 40 °C for 5 h. The resulting product was collected by an external magnetic field prior to being washed with ethanol several times, with DI water, and acetone, and it was dried with a freeze-dryer. In the next step, 200 mg of Fe_3_O_4_-SiO_2_ was mixed with (3-Aminopropyl) triethoxysilane (APTES) (2 mL); then, the mixture was refluxed in dry toluene (100 mL) at 70 °C for 24 h, and the obtained amino-functionalized MNPs were isolated from the reaction mixture with a permanent external magnet before being washed with ethanol and DI-water several times; then, they were dried with a freeze-dryer [34,35].

### 4.5. Synthesis of SPION@SiO_2_@HPG

First, 100 mg of silica-coated magnetic nanoparticles were dispersed in 2 mL of a saturated potassium methoxide solution in methanol using bath sonication for 30 min, and the solution was stirred at room temperature for one h and refluxed at 80 °C for 2 h. In the end, the magnetic nanoparticles were separated with a magnet, washed with dry methanol three times, and dried with a freeze-dryer. Then, 2 mL glycidol was added to these deprotonated nanoparticles drop-wise for 15 min at 100 °C, and this mixture was refluxed at this temperature for four h. Then, it was cooled, and the contents were dissolved in methanol. Consequently, the product was separated with a magnet prior to being washed with methanol under sonication several times. The obtained product was dried with a freeze-dryer overnight in order to obtain the HPG-grafted silica-magnetic nanoparticles [42].

### 4.6. Folic Acid-Targeting of Nanoparticles

**Figure 10:**
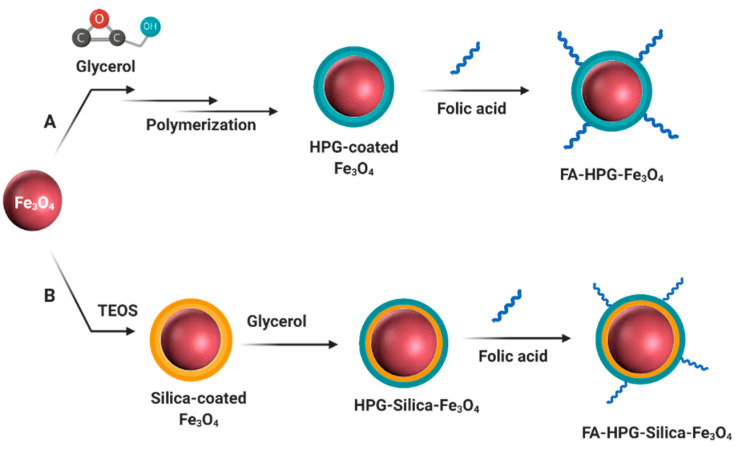
Synthesis and preparation of folic acid decorated nanoparticles.

Conjugation of folic acid on Fe_3_O_4_@HPG and Fe_3_O_4_@SiO_2_@HPG was done according to the previous report [45]. Briefly, 5 mg FA and 100 mg Fe_3_O_4_ coated nanoparticles were added to 5 mL dry DMSO, and the mixture was sonicated for 2 min. Subsequently, 1.53 mg DMAP and 2.96 mg DCC were added to this mixture as intermediator. The resulting brown suspension was bath sonicated for 2 min and stirred at 50 °C for 36 h by magnetic stirring. After completing the process, the solution was dialyzed against DI-water (dialysis membrane 12–14 kDa) to remove DMAP, DCC, and unbounded FA. Finally, the solution was dried with a freeze-drier (Figure 10).

### 4.7. Characterization Techniques

FT-IR spectra were obtained in a transmission mode (JASCO, FT-IR-6300 (400–4000 cm^−1^), Tsukuba, Japan) to determine the surface modification of nanoparticles. The size and core-shell structure of nanoparticles were observed with transmission electron microscopy (JEOL JEM-ARM200CFEG UHR-TEM (Peabody, MA, USA) (equipped with STEM, Cs corrected STEM, EDS, Gatan Quantum GIF and Digital CCD Camera)). Magnetization measurements were carried out on a certain amount of ferrofluid by employing a vibrating sample magnetometer (VSM; MDKFT, Kosar Kashan Magnetics Corporation, IR, Kashan, Iran). This system specifies the amount and magnetization behavior of the sample as a function of the changes in the intensity of the constant magnetic field applied.

### 4.8. MTT Assay

The cytotoxicity of nanoparticles was determined using the MTT colorimetric assay. In this test, MCF-7 cell line, as a type of cancer cell with overexpression of FA receptor, was seeded in 96-well plates at a density of 5000 cells/well in 100 µL of medium, and it was incubated overnight at 37 °C in 5% CO_2_. After 24 h of incubation, the media of all wells were replaced with 100 µL of fresh media, holding different concentrations (25, 50, 100, and 200 µg/mL) of nanoparticles. All concentrations were replicated in three wells. After 24 and 48 h of incubation, the media were removed, and cells were washed with PBS twice. Then, 100 µL of pure medium was added to each well, followed by the addition of 10 µL of MTT solution (5 mg/mL in PBS), and the plates were incubated for four h at 37 °C. Then, the media were replaced by 100 µL DMSO and the plates were incubated for another 1 h. Finally, the wells absorbance was measured at 490 nm with a microplate reader (Bio-Rad Laboratories, Inc., Hercules, CA, USA), in order to determine the viability of the cells.

### 4.9. In Vitro MR Imaging

MR imaging was carried out with a clinical 1.5 T MR Imager and a high-resolution head coil. MCF-7 cell line (250,000 cells per well) was incubated with different concentrations of SPION@HPG, SPION@HPG-FA, SPION@SiO_2_@HPG and SPION@SiO_2_@HPG-FA (0.0625, 0.125, 0.250 and 0.500 mM Fe) for 4 h. Then, the cells were washed with PBS twice to remove non-attached nanoparticles. The attached cells were then separated by adding trypsin and re-suspending in 1.5 mL gelatin. Determination of the *T*_2_ relaxation times was carried out by a multi-echo spine-echo sequence (repetition time (TR): 3000 ms; echo time (TE): 18–144 ms), slice thickness of 20 mm, and the field of view of 160 mm. The relaxation times for each sample were calculated by using in-house software (MATLAB 2016b) by non-linear least-squares fitting of appropriate exponential functions. Transverse relativities ($r_{2}$) were calculated as the slope of the lines fitted by linear regression analysis (Microsoft EXCEL) to relaxation rates (1/*T*_2_).

## 5. Conclusions

In this research, the coating and targeting of iron oxide nanoparticles were compared and evaluated on their relaxivity, by conjugating HPG and SiO_2_@HPG as coating agents and the FA ligand as a targeting agent on the surface of nanoparticles. The findings of the research revealed that the amount of magnetic saturation for SiO_2_@HPG coating nanoparticles was higher than HPG coating. SiO_2_@HPG-FA targeted nanoparticles showed a high relaxivity of 23.25 mM^−1^S^−1^ compared to non-targeted SiO_2_@HPG nanoparticles. Moreover, HPG targeted nanoparticles have an $r_{2}$ value of about 0.131 mM^−1^S^−1^—considerably lower than SiO_2_@HPG@FA nanoparticles. Therefore, FA ligand could award this nanoparticle with targeting specificity to the tumor cells via receptor-mediated endocytosis. With these promising properties, Fe_3_O_4_@SiO_2_@HPG-FA nanoparticles have good potential to be used as MRI contrast agents in cells that have over-expression of folate receptors. However, future studies should be devoted to carry out in vivo test to understand its potential bio-applications.

## Figures and Tables

**Table 1:** In vitro Transverse relativities ($r_{2}$ ) of MRI contrast agents.

	$r_{2}\;[{\mathrm{mM}}^{-1}S^{-1}]$
Fe_3_O_4_@SiO_2_@HPG	8.87
Fe_3_O_4_@SiO_2_@HPG-FA	23.25
Fe_3_O_4_@HPG	0.029
Fe_3_O_4_@HPG-FA	0.131
